# Citability of Original Research and Reviews in Journals and Their Sponsored Supplements

**DOI:** 10.1371/journal.pone.0009876

**Published:** 2010-03-24

**Authors:** Leslie Citrome

**Affiliations:** 1 Department of Psychiatry, New York University School of Medicine, New York, New York, United States of America; 2 The Nathan S. Kline Institute for Psychiatric Research, Orangeburg, New York, United States of America; University of Illinois at Champaign-Urbana, United States of America

## Abstract

**Background:**

The contents of pharmaceutical industry sponsored supplements to medical journals are perceived to be less credible than the contents of their parent journals. It is unknown if their contents are cited as often. The objective of this study was to quantify the citability of original research and reviews contained in supplements and compare it with that for the parent journal.

**Methodology/Principal Findings:**

This was a cohort study of 446 articles published in the *Journal of Clinical Psychiatry* (JCP) and its supplements for calendar years 2000 and 2005. The total citation counts for each article up to October 5, 2009 were retrieved from the ISI Web of Science database. The main outcome measure was the number of citations received by an article since publication. Regular journal articles included 114 from calendar year 2000 and 190 from 2005. Articles from supplements included 90 from 2000 and 52 from 2005. The median citation counts for the 3 years post-publication were 10 (interquartile range [IQR], 4–20), 14 (IQR, 8–20), 13.5 (IQR, 8–23), and 13.5 (IQR, 8–20), for the 2000 parent journal, 2000 supplements, 2005 parent journal, and 2005 supplements, respectively. Citation counts were higher for the articles in the supplements than the parent journal for the cohorts from 2000 (p = .02), and no different for the year 2005 cohorts (p = .88). The 2005 parent journal cohort had higher citation counts than the 2000 cohort (p = .007), in contrast to the supplements where citation counts remained the same (p = .94).

**Conclusions/Significance:**

Articles published in JCP supplements are robustly cited and thus can be influential in guiding clinical and research practice, as well as shaping critical thinking. Because they are printed under the sponsorship of commercial interests, they may be perceived as less than objective. A reasonable step to help improve this perception would be to ensure that supplements are peer-reviewed in the same way as regular articles in the parent journal.

## Introduction

Supplements to biomedical journals can be used as a forum to highlight a particular disease state or intervention. They are often sponsored by a commercial enterprise with a stake in the topic being discussed. For example the sponsor can be a pharmaceutical company wishing to promote their medications. Usually the supplement is funded through an unrestricted grant and CME credits may or may not be provided. The fee charged for a supplement is not usually made explicit, but a figure of $50,000 for up to 60 pages has been publically noted for supplements to the journal *Chest* [1]. Recommendations have been made regarding the role of the journal editor, selection of a supplement editor, disclosure of funding source, and the role of the funding organization [2]. Individual journals have published their policies and commentaries on this topic in editorials [1], [3]–[8].

Although the peer review process (if any) often differs from that of the parent journal (as will be described in our case example), it is not unusual for the contents of these journal supplements to be themselves cited in scholarly works. Controversy arises when the contents of the supplements are perceived to be biased in favor of the sponsor [9]–[20]. This has also led to spirited discussions in the internet's “blogosphere” [21]. The topic of journal supplements has figured prominently in testimony regarding promotional activities of pharmaceutical companies [22]. Supplements at times are derived from “consensus conferences” which some have opined is a form of drug promotion [23]. In rare cases, distribution of supplements has been withheld from certain subscribers because of drug regulatory concerns [24].

Although the quality of articles published in journal supplements have been compared with the quality of those published in the parent journal, as reported by Rochon and colleagues in 1994 [25], what remains unanswered is how often are articles in supplements actually cited. The aim of this study is to contrast the citation rates for articles appearing in the 2000 and 2005 issues of the Journal of Clinical Psychiatry and its supplements, using citation profiles in the ISI Web of Science database [26].

## Methods

The *Journal of Clinical Psychiatry* (JCP) was selected as the prototypical biomedical journal that regularly publishes supplements. JCP is a peer-reviewed psychiatry specialty journal published monthly by Physicians Postgraduate Press, Inc. [27]. JCP is the official publication of the American Society of Clinical Psychopharmacology [28] and is provided to its members as a benefit; however the only regular content that is directly attributable to activities of this society is a column, the “ASCP Corner.” According to JCP's information for media planners [29], the journal has 35,613 subscribers and is the 3rd most cited psychiatric journal in the world with a journal impact factor of 5.533 as of December 2008, and ranks highest in the mean total number of office- and hospital-based issue readers of psychiatric journals according to a June 2008 Focus® Readership Study. Most subscribers receive the journal free of charge if they are designated as psychiatric clinicians in provider databases such as the American Medical Association's Masterfile. Reprints of individual articles and supplements can also be widely distributed as part of marketing and informational campaigns conducted by the pharmaceutical industry. JCP's masthead reads “Our primary mission is to provide lifelong learning for the physician through evidence-based, peer-reviewed scientific information about the diagnosis and treatment of behavioral health and neuropsychiatric disorders” [30]. Articles in the parent journal are a mixture of research reports, including reports of randomized controlled trials, and reviews. Articles in the supplements are usually reviews. Articles are indexed in MEDLINE/PubMed, EMBASE/Excerpta Medica, Psychological Abstracts, Current Contents, Science Citation Index, Hospital Literature Index, Biological Abstracts, Cumulative Index to Nursing and Allied Health Literature, International Nursing, PsycINFO, Chemical Abstracts, Adolescent Mental Health Abstracts, Alcohol and Alcohol Science Problems Database, and the Social Sciences Citation Index. Papers submitted to JCP undergo the usual and customary peer review process by expert consultants [31], i.e., typically two or more independent reviewers are charged with examining the quality of the study or review and its potential importance to the field. Supplements to JCP undergo a different review process as disclosed in the supplement, and include a planning session (telephone or live) at which the authors have the opportunity to comment on each other's presentations/submissions, a prepublication review by the pre-designated Chair of the activity for accuracy and fair balance (the Chair often writing an introduction as well as one or more articles in the supplement), and a prepublication review for fair balance by a reviewer from the CME Institute of Physicians Postgraduate Press, Inc [32]. The role of the journal editor in the production of the supplement is not explicitly stated.

The tables of contents for the JCP issues from calendar years 2000 and 2005, including supplements, were obtained electronically from the JCP Web site [27]. Four cohorts of articles were identified: JCP articles from 2000, JCP supplement articles from 2000, JCP articles from 2005, and JCP supplement articles from 2005. Included were all articles in the parent journal or supplement for which an on-line abstract is available; excluded from further consideration were publisher's notes, editorials, commentaries, introductions to special sections, letters to the editor, columns, book reviews, or any other items not accompanied by an on-line abstract. Excluded from further consideration from the supplements were any introductory or concluding remarks or discussion for which an on-line abstract is not available. The numbers of citations to the included articles (citable articles) were determined by querying the ISI Web of Science database [26] for January 1, 2000 through October 5, 2009 for the articles published in 2000, and for January 1, 2005 through October 5, 2009 for the articles published in 2005. The ISI database captures citations from scholarly journals, books, book series, reports, and conference proceedings. No constraints were used. No sample size calculation was made prior to the data extraction. Mean, median, and range of number of citations for each cohort of articles were calculated.

The citation counts for each cohort are described using summary statistics. Compared were the number of citations for the parent journal versus its supplements for the first 3 complete calendar years post-publication, i.e. 2001 through 2003 for the 2000 cohorts and 2006 through 2008 for the 2005 cohorts. Also compared were the 2000 versus the 2005 cohorts to detect any possible changes in citation counts. Because citation counts do not follow a Gaussian distribution and the sample size is small (particularly for the 2005 supplement cohort), nonparametric testing using the two independent samples Wilcoxon Rank Sum test was performed with a threshold of p<.05 for significance (using software provided at http://www.socr.ucla.edu/htmls/ana/TwoIndependentSampleWilcoxonRankSum_Analysis.html). As this analysis is principally descriptive, no adjustment was made for multiple comparisons. *Post hoc* exploration was conducting comparing the proportion of articles that generated ≥25 and ≥40 citations during the first 3 complete calendar years post-publication, and significance for this was tested by Fisher's exact test (using software provided at http://www.openepi.com/Menu/OpenEpiMenu.htm). The threshold of ≥25 characterizes articles that are at least in the top quartile.

## Results

**Figure 1:**
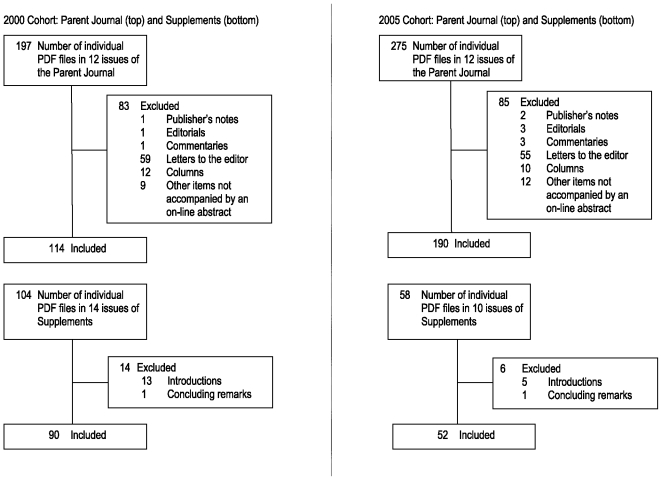
Flow Diagram of Articles in Each Cohort.

In both 2000 and 2005, 12 regular issues of JCP were published in each year. In 2000, 14 supplements were published, 9 of which were designated as CME activities, with instructions, posttest, registration and evaluation. In 2005, 10 supplements were published, with 7 containing information on obtaining CME credit. Figure 1 describes the number of individual PDF files available electronically and the number of eligible articles included in each cohort. Table 1 contains the citation counts for each cohort, including mean ± SD, median, and full range, for all years where data is available. The interquartile range for the citation counts 3 years post-publication were 4–20, 8–20, 8–23, and 8–20, for the 2000 parent journal, 2000 supplements, 2005 parent journal, and 2005 supplements, respectively.

**Table 1:** Citation counts for articles contained in the Journal of Clinical Psychiatry and supplements, 2000 and 2005.

Year	Citations per Article							
	2000 Cohort				2005 Cohort			
	Parent Journal (N = 114 in 12 issues)		Supplements (N = 90 in 14 issues)		Parent Journal (N = 190 in 12 issues)		Supplements (N = 52 in 10 issues)	
	median, range	mean ± SD	median, range	mean ± SD	median, range	mean ± SD	median, range	mean ± SD
2000	0, 0–4	0.52±.96	0, 0–6	0.83±1.17	–	–	–	–
2001	2, 0–21	3.05±3.25	3, 0–14	3.40±2.99	–	–	–	–
2002	3.5, 0–50	5.29±6.59	6, 0–20	5.80±3.78	–	–	–	–
2003	3.5, 0–50	5.89±6.76	6, 0–16	6.04±3.97	–	–	–	–
2004	4, 0–37	5.27±6.10	4.5, 0–28	5.63±4.68	–	–	–	–
2005	3, 0–32	5.26±5.89	5, 0–33	5.27±4.62	0, 0–11	0.73±1.53	0, 0–5	0.79±1.13
2006	3, 0–26	4.68±4.55	4, 0–26	4.34±4.00	3, 0–25	4.82±4.70	3, 0–16	3.64±3.06
2007	3, 0–23	4.06±4.46	3. 0–31	4.46±4.60	5, 0–40	6.52±6.49	4, 0–19	6.00±4.64
2008	2, 0–24	4.00±4.80	3, 0–39	4.12±5.36	5, 0–30	6.72±6.06	5, 0–42	6.54±6.33
2009^a^	2, 0–12	2.41±2.54	1.5, 0–18	2.31±2.85	3, 0–25	4.16±4.42	3, 0–38	3.83±5.69
All years	27.5, 0–264	40.44±39.33	39, 4–206	42.21±29.62	16.5, 0–111	22.94±19.80	16, 2–100	20.79±16.55
3 years post-publication b	10, 0–116	14.22±15.42	14, 1–49	15.24±8.86	13.5, 0–85	18.05±15.44	13.5, 2–62	16.17±11.78

a To October 5, 2009.

b 2001–2003 for the cohorts from 2000; 2006–2008 for the cohorts from 2005.

Table 2 provides the results of the comparisons between the 4 cohorts.

**Table 2:** Nonparametric analyses of citation counts for the first 3 complete calendar years post-publication a b.

Comparison	z-score	p-value
Parent journal vs supplements, 2000 cohorts	2.309	.02
Parent journal vs supplements, 2005 cohorts	.145	.88
Parent journal, 2000 vs 2005	2.679	.007
Supplements, 2000 vs 2005	.074	.94

a 2001–2003 for the cohorts from 2000; 2006–2008 for the cohorts from 2005.

b As calculated at http://www.socr.ucla.edu/htmls/ana/TwoIndependentSampleWilcoxonRankSum_Analysis.html; 2-tail p-value provided.

Table 3 provides the proportion of articles that generated ≥25 and ≥40 citations during the first 3 complete calendar years post-publication.

**Table 3:** Proportion of articles that have generated ≥25 and ≥40 citations during the first 3 complete calendar years post-publication a.

Category	Proportion									
	2000 Cohort					2005 Cohort				
	Parent Journal (N = 114 in 12 issues)		Supplements (N = 90 in 14 issues)		p-value b	Parent Journal (N = 190 in 12 issues)		Supplements (N = 52 in 10 issues)		p-value b
	n	%	n	%		n	%	n	%	
≥25 citations	14	12.3%	13	14.4%	0.80	45	23.7%	8	15.4%	.27
≥40 citations	4	3.5%	1	1.1%	0.53	20	10.5%	2	3.8%	.21

a 2001–2003 for the cohorts from 2000; 2006–2008 for the cohorts from 2005.

b Fisher's exact test (as calculated at http://www.openepi.com/Menu/OpenEpiMenu.htm); 2-tail p-value provided.

## Discussion

Articles published in supplements in JCP are often cited when compared to the parent journal. The median number of citations from the 2000 cohorts appears to favor the supplements; although 5 years later there were no differences in the median number of citations in the 2005 cohorts. When adjusting for time by limiting the citation counts to the subsequent 3 calendar years after publication, the median number of citations for both the parent journal and the supplements were higher for the 2005 cohorts compared to the 2000 cohorts, but with a greater relative increase noted for the parent journal. The proportions of highly cited articles (articles that have generated at least 25 or 40 citations) were no different statistically between the parent journal and the supplements although a numerically higher proportion was consistently observed in favor of the parent journal for the 2005 cohort.

The mean number of citations per citable article shown in table 1 can be viewed as a “citability index” and is similar in concept to the journal impact factor [33]. As with the journal impact factor, there is wide variability in citation rates for individual articles no matter the overall citability index [34].

The ISI database used in this study does not include all possible citation sources. For example, the Google Scholar database [35] can yield higher numbers of citations [36]. However it is doubtful that these additional sources would skew either towards citations to the parent journal or to its supplements enough to make a difference in the overall findings presented here.

A caveat is that the results of this bibliometric analysis of articles from JCP may not be generalizable to other journals that principally publish research reports and/or to those journals that do not regularly issue supplements. In general, multi-specialty and specialty journals can have diverse aims, scope, and editorial standards. Not all supplements from all journals are financed by pharmaceutical or medical device manufacturers. Some journals may already have in place a rigorous peer-review process for their supplements. This study should be replicated using another journal which makes much use of supplements, perhaps in a field of specialty other than psychiatry.

Citation counts by themselves may not be necessarily reflective of the impact an article has on actual day-to-day clinical practice; citations in the literature are done primarily by academics and not ordinarily by community practitioners. Moreover, the number of citations an article receives may not be representative of the number of readers who have accessed the article. An article may be clinically influential yet not often cited. Other metrics such as number of downloads from a website, or the number of reprints ordered by commercial interests, may provide additional information regarding how many persons have actually obtained a copy of the article (or supplement).

Whether or not citing articles from supplements is good practice is not addressed by this analysis. Citation rates may not be related to the quality of the cited articles. It is possible that the highly cited articles, regardless of where they were published, may contain information that other authors may have found useful to support their arguments. It is also possible that an article is cited to identify methodological shortcomings and to show how the citing study intends to rectify those limitations. A limitation of this study is that the individual quality of each cited report was not assessed, and no distinction was made between reports of randomized controlled trials, other types of original reports, and reviews. The contents of the supplements were not further scrutinized regarding funding source or potential biases in the articles themselves. There is some published work regarding quality of articles in journal supplements versus the parent publication, but all of it predates this decade [25], [37], [38]. Also unexplored are whether articles published in supplements are preferentially cited by other articles that have been sponsored in some way.

Of additional interest is the impact of self-citation (i.e. citing one's previous publication in a new publication). Self-citation was reported in a bibliometric analysis of articles about diabetes mellitus in 170 clinical journals published in 2000 [39]. Nearly one-fifth of all citations were author self-citations [39]. However in that report, original articles had twice the proportion of author self-citations compared with review articles. Assuming this observation is generalizable to the JCP, because supplements usually contain reviews and the parent journal usually contains a high proportion of research reports, the impact of author self-citation on differential citation rates between supplement and parent may be difficult to interpret. Another issue is that of journal self-citation, i.e. when publications in a journal cite previous publications in the same journal [40]. Journal self-citation can have a positive effect on a journal's impact factor [41], and can potentially affect citation rates to articles in supplements and their parent journal.

Unaddressed in this analysis is the issue of ghost authorship [42], [43] and ghost management [44] of supplements. “Ghost writing” was described in 1934 [45] and in that report the author recommended that the assistance of medical writers be acknowledged. Several decades later it is apparent that this acknowledgement is not always made. It may be that supplements may be fertile ground for this behavior, given the different degree of editorial scrutiny applied to supplements compared to their parent journals.

### Conclusions

Articles published in JCP supplements are robustly cited, with many cited more often than many articles published in the parent journal. Thus articles in supplements may be quite influential in guiding clinical and research practice, as well as shaping critical thinking. However, articles in supplements are not subject to the same rigor of peer review as the parent journal and because they are printed under the sponsorship of commercial interests, they may be perceived as less than objective. A reasonable step to help improve this perception would be to ensure that supplements are peer-reviewed in the same way as regular articles in the parent journal.
